# Grafted Teeth

**Published:** 1884-07

**Authors:** 


### GRAFTED TEETH.

TRANSPLANTING sound teeth from the jaws of healthy persons who could spare them to those who needed them has been practised by advanced dentists for some time. The mode was as follows: The individual with the superfluous sound tooth and he with the dedayed molar were mated together, and the freshly drawn good tooth immediately placed in the cavity made by extracting the other. But it often happens to be necessary to remove a sound tooth from a patient at a time when no person of whom the dentist had any knowledge needed just such a one. It would therefore be lost, for only "living" teeth could be made to grow in a strange mouth, and they died very soon after being torn from their parent gums. Teeth are "living" so long as the membrane covering the roots has any vitality. It has been a problem of great interest to dentists throughout America to devise some means by which the sound extracted teeth could be kept alive indefinitely, until they should be needed, and to a San Francisco dentist belongs the honor of solving the problem. Dr· W. J. Younger, says the *Call*, has been conducting a series of experiments, which have resulted in the discovery of a means of preserving the life of extracted teeth. It is nothing more or less than "grafting" it as soon as it is drawn, upon the engorged comb of a healthy rooster, and leaving it there properly secured until it is wanted. Then it is cut away, the cock being placed under the influence of chloroform, washed, and everything removed down to the membrane, and placed in the freshly made cavity where it is needed. A representative of the *Call* was permitted recently to examine the mouth of a gentleman in which there was a tooth that had been planted there a week or so before, and which was apparently as firm as those which had always been there. It had been kept alive on a cock's comb for ten days, and had been taken from the mouth of a young lady, whose looks were benefitted by the removal.

